# The Cytolytic Activity of Vaginolysin Strictly Depends on Cholesterol and Is Potentiated by Human CD59

**DOI:** 10.3390/toxins7010110

**Published:** 2015-01-13

**Authors:** Milda Zilnyte, Česlovas Venclovas, Aurelija Zvirbliene, Milda Pleckaityte

**Affiliations:** Institute of Biotechnology, Vilnius University, Graičiūno 8, Vilnius LT-02241, Lithuania; E-Mails: milda.zilnyte@bti.vu.lt (M.Z.); ceslovas.venclovas@bti.vu.lt (Č.V.); aurelija.zvirbliene@bti.vu.lt (A.Z.)

**Keywords:** cytolysin, vaginolysin, hCD59, cholesterol, liposomes, pore

## Abstract

*Gardnerella vaginalis* produces cytolysin vaginolysin (VLY), which has been suggested to be a contributor to bacterial vaginosis pathogenesis. VLY along with intermedilysin (ILY) from *Streptococcus intermedius* have been attributed to a group of cholesterol-dependent cytolysins (CDCs) whose pore-forming activity depends on human CD59 (hCD59). Here, we show that different types of cells lacking hCD59 are susceptible to VLY-mediated lysis, albeit to different extents. We analyze the effects of both hCD59 and cholesterol on VLY cytolytic activity. We show that VLY binds to cholesterol-rich membranes of non-human cells, while VLY with an impaired cholesterol recognition site retains binding to the hCD59-containing cells. We further demonstrate that cholesterol binding by VLY is sufficient to trigger the formation of oligomeric complexes on cholesterol rich-liposomes lacking hCD59. Thus, VLY may induce cell lysis following two alternative pathways. One requires only cholesterol and does not depend on hCD59. The second pathway involves hCD59 contribution similarly to ILY*.* Apparently, under physiological conditions VLY acts in the most effective way by accepting the assistance of hCD59.

## 1. Introduction

Cholesterol-dependent cytolysins (CDCs) comprise a class of structurally related bacterial pore-forming toxins produced by a large number of Gram-positive pathogens [1,2]. CDCs have been considered as virulence factors promoting bacterial invasion and infection [2]. Secreted as soluble monomers, CDCs bind to host cell membranes and form large, circular oligomeric complexes on the surface of the membrane and then insert into the bilayer to form aqueous pores [3,4,5,6,7]. The initial stage of the pore formation is the binding of CDC to the eukaryotic cell membrane using cholesterol as a receptor [4,5,6,7,8]. CDCs employ a cholesterol-binding motif comprising a threonine-leucine pair located in domain 4 of the molecule [9]. The mechanism of pore formation has been most extensively studied for perfringolysin (PFO) from *Clostridium perfringens*. It was proposed that membrane-bound PFO monomers undergo a structural transition leading to the formation of monomer-monomer contacts [3,10,11]. This structural transition takes place in domain 3 of PFO and involves the rotation of the loop comprised of β5 and α1 away from β4, enabling the membrane-bound monomers to oligomerize via the interaction of β1 and β4 strands of adjacent monomers [3,11].

A distinct group of CDCs emerged after the discovery that intermedilysin (ILY) from *Streptococcus intermedius* binds to surface-located human complement glycoprotein CD59 (hCD59) rather than to cholesterol [12,13]. Human CD59 is a glycosyl-phosphatidylinositol (GPI)-anchored membrane protein that blocks the formation of the complement membrane attack complex (MAC) by binding complement proteins C8α and C9 [14,15]. Binding to human CD59 drives conformational changes in the domain 3 of the toxin leading to the oligomerization of the membrane-bound monomers, while cytolytic activity of ILY remains fully dependent on the membrane cholesterol [13,16]. The dependence on hCD59 makes ILY specific for human cells: animal erythrocytes were shown not to be susceptible to ILY [17]. Subsequent studies have demonstrated that only a few other CDCs, namely vaginolysin (VLY) from *Gardnerella vaginalis* and lectinolysin from *Streptococcus mitis* use hCD59 as their receptor [18,19].

*G. vaginalis* has been identified as a prevailing inhabitant of the vaginal tract of women diagnosed with bacterial vaginosis (BV) [20]. VLY secreted by *G. vaginalis* is able to lyse human erythrocytes and human vaginal epithelial cells [18,21]. The toxin activates epithelial p38 mitogen-activated protein kinase pathway and it is probably connected with fluctuations of the IL-8 level in BV patients [18]. The VLY secretion level varies among *G. vaginalis* strains and may correlate with the severity of BV condition [22].

It was demonstrated that VLY is selective for human cells as mouse and other non-human erythrocytes are markedly less susceptible to VLY-mediated lysis [18,21,23]. Moreover, the monoclonal antibody-mediated blockade of hCD59 on the surface of human erythrocytes suspends the VLY-induced cell lysis [18]. However, recent findings based on the reconstitution of VLY into artificial tethered bilayer membranes (tBLMs) lacking hCD59 demonstrated that hCD59 is not an essential factor for the VLY hemolytic activity [24]. VLY expressed the pore-forming ability on a phospholipid membrane in the absence of human complement protein while the requirement for the cholesterol was strictly unconditional [24].

In this paper we analyzed the effects of both hCD59 and cholesterol on VLY cytolytic activity. We demonstrate that VLY is able to use both hCD59 and cholesterol as receptors. Furthermore, we show that, in contrast to ILY [13,16], cholesterol binding by VLY is sufficient to trigger the formation of oligomeric complex and lead to VLY-mediated lysis of the cells lacking hCD59.

## 2. Results

### 2.1. VLY Cytolytic Activity on Human and Non-Human Cells

**Figure 1:**
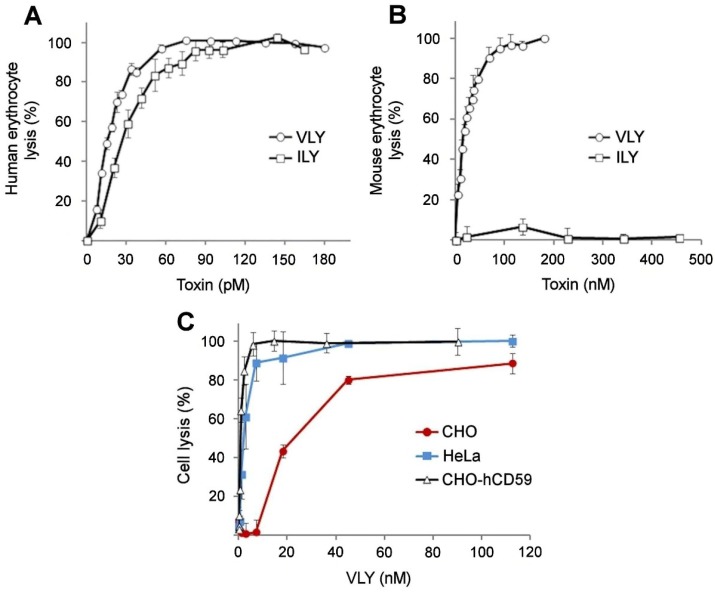
Hemolytic activity of recombinant vaginolysin (VLY) and intermedilysin (ILY) using human (**A**) and mouse (**B**) erythrocyte suspension; (**C**) VLY cytolytic activity on CHO, CHO-hCD59 and HeLa cells.

Previously determined dependence of VLY and ILY pore-forming mechanisms on hCD59 [12,13,16,18] prompted us to compare hemolytic and cytolytic activities of these toxins using human and non-human cells. Human erythrocytes being a rich source of hCD59 were nearly equally susceptible to VLY and ILY-mediated lysis (HD_50_ = 10 ± 1 pM and HD_50_ = 26 ± 1.5 pM, respectively) (Figure 1A). However, mouse erythrocytes were affected differently by VLY and ILY. Even high concentrations of ILY (HD_50_ > 2000 nM) did not lyse mouse red blood cells (Figure 1B). In contrast, they were susceptible to the VLY treatment, although the amount of VLY required to lyse mouse erythrocytes was substantially higher (HD_50_ = 16 ± 1 nM) than that required to lyse human erythrocytes (Figure 1A,B).

Erythrocytes are not the single VLY target under physiological conditions [25]. Therefore, we assayed VLY cytolytic activity on human (HeLa) and non-human (CHO) cell lines derived from human and non-human tissues. Human epitheloid HeLa cells expressing the hCD59 protein on their surface were lysed by VLY (Figure 1C). CHO cells and CHO cells expressing hCD59 (CHO-hCD59) were also susceptible to VLY-mediated lysis but to a different degree (Figure 1C). Calculated HD_50_ values demonstrate that the amount of VLY required to lyse CHO-hCD59 cells is about 32-fold less (HD_50_ = 0.7 ± 0.2 nM) than that required to lyse CHO cells (HD_50_ = 23 ± 5 nM) and are comparable to the amount applied for HeLa cell lysis (HD_50_ = 2.3 ± 0.4 nM). Again, these results contrast starkly with observations involving ILY. Even at high concentrations of ILY, cells that do not express hCD59 on their surface were not subjected to ILY-mediated lysis [17]. To understand the differences in VLY and ILY activities, we examined VLY ability to bind to CHO and CHO-hCD59 cells and form oligomer complexes on cholesterol-rich liposomes.

### 2.2. VLY Binding to CHO and CHO-hCD59 Cells

VLY binding to CHO cells and CHO cells expressing hCD59 was examined by flow cytometry. We constructed a VLY double mutant with substituted two absolutely conserved glycines (G308A and G309A) at the junction of β4 and β5 strands in domain D3 [3]. These substitutions were aimed at trapping VLY in the monomer-locked (ML) state similarly to PFO, in which the mutation of the corresponding glycine pair blocks the disengagement of β5 from β4, necessary to convert monomers into oligomers [3]. The VLY mutant arrested in the monomer-locked state (VLY(ML)) was purified and assayed for the hemolytic activity on human erythrocytes and for the ability to bind to immobilized cholesterol on PVDF membrane (Figure S1). VLY(ML) lost the pore-forming activity (Figure S1B), but retained its ability to bind to membrane-immobilized cholesterol (Figure S1A). We also constructed another VLY double mutant T474G·L475G (VLY-TL). The rationale of converting the Thr-Leu pair to glycines was based on the previous reports demonstrating an essential role of these amino acid residues in cholesterol binding [9,24]. The VLY-TL mutant showed no hemolytic activity on human erythrocytes and did not bind to immobilized cholesterol (Figure S1). Detailed characteristics of VLY mutants are presented in Table 1.

**Table 1:** Characteristics of vaginolysin (VLY) mutants.

VLY variants	Cholesterol binding (nmol cholesterol) ^a^	Oligomer formation detected by denaturing agarose gel electrophoresis	HD_50_ (pM) ^b^
VLY	0.25	+	10 ± 1
VLY(ML): G308A·G309A	0.25	-	>2000
VLY-TL: T474G·L475G	>65	-	>2000
Y165A	0.25	+	18 ± 1
I306P	0.25	-	>1000
A304P·I306P	1.02	-	>1000
V305P	0.25	+	17 ± 5

^a^ Visually detected spot on the dot blot, which corresponds to minimal amount of PVDF-membrane immobilized cholesterol required for binding of VLY and its derivatives. ^b^ HD_50_ was defined as the concentration of VLY and its derivatives required to lyse 50% of human erythrocytes as described in Experimental section. VLY: vaginolysin.

VLY and its derivatives preincubated with the cells were probed with monoclonal antibodies (MAbs) against VLY [21]. The anti-VLY MAbs were detected with a fluorescently labeled secondary antibody allowing cell-bound VLY to be detected by flow cytometry. Two primary anti-VLY MAbs 9B4 and 21A5 were used [21]. Competitive ELISA and epitope mapping revealed that MAbs 9B4 and 21A5 recognize distinct antigenic sites of VLY [21]. The reactivity of these MAbs with each VLY mutant was characterized by their apparent *K*_d_ values determined by an indirect ELISA (Table 2). The binding affinity of MAb 21A5 with each mutant was lower than that of MAb 9B4. The CHO cell line transfected with the control vector pFUSE-hIgG1 and non-transfected CHO cell line did not show any discrepancies in VLY binding (data not shown), therefore subsequent experiments were performed using non-transfected CHO cells unless otherwise stated.

**Figure 2:**
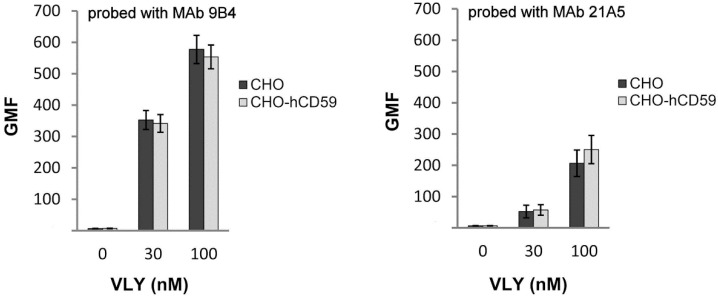
Binding of native vaginolysin (VLY) to CHO and CHO-hCD59 cells probed with monoclonal antibodies (MAbs) 9B4 and 21A5 detected by flow cytometry. The figures are representative of experiments performed at least in triplicate. GMF: geometric mean fluorescence.

The flow cytometry revealed that native VLY binds to CHO and CHO-hCD59 (Figure 2).

**Table 2:** Quantitative measurement (*K*_d_) of the affinity of anti-VLY monoclonal antibodies (MAbs) with vaginolysin (VLY) mutants.

MAbs	Native VLY	VLY(ML)	VLY-TL
9B4	3 × 10^−10^ M	2.2 × 10^−10^ M	2.5 × 10^−10^ M
21A5	2.7 × 10^−10^ M	0.9 × 10^−9^ M	0.7 × 10^−9^ M

**Figure 3:**
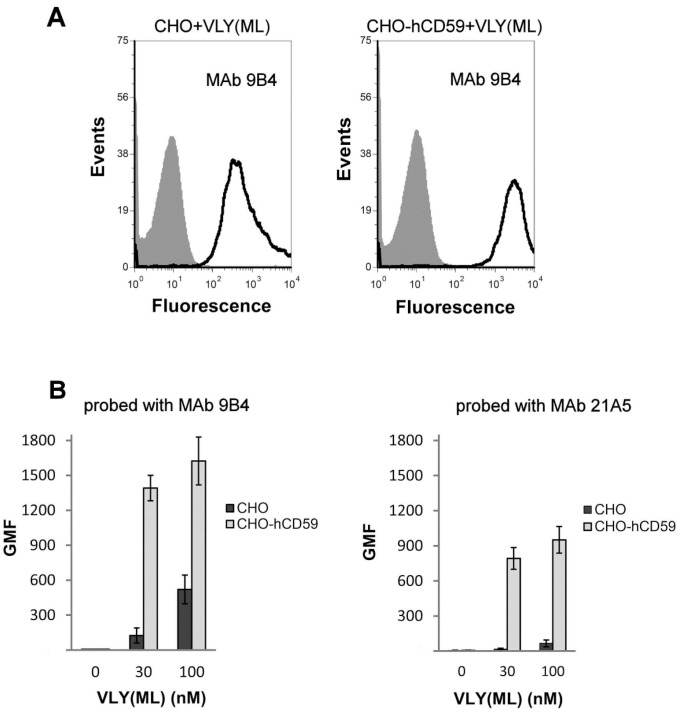
(**A**) The overlay histograms represent binding profile of vaginolysin mutant VLY(ML) to CHO and CHO-hCD59 cells probed with monoclonal antibody (MAb) 9B4; (**B**) Binding of mutant protein VLY(ML) to CHO and CHO-hCD59 cells probed with MAbs 9B4 and 21A5 detected by flow cytometry. The figures are representative of experiments performed at least in triplicate. GMF: geometric mean fluorescence.

The VLY(ML) mutant bound to non-transfected CHO cells (Figure 3), but the relative fluorescence, indicating the number of bound VLY molecules, was approx. 3.5-fold lower as compared to that of CHO-hCD59 binding (Figure 3B). The same binding tendency of the VLY(ML) mutant to both cell types was also observed using MAb 21A5 (Figure 3).

**Figure 4:**
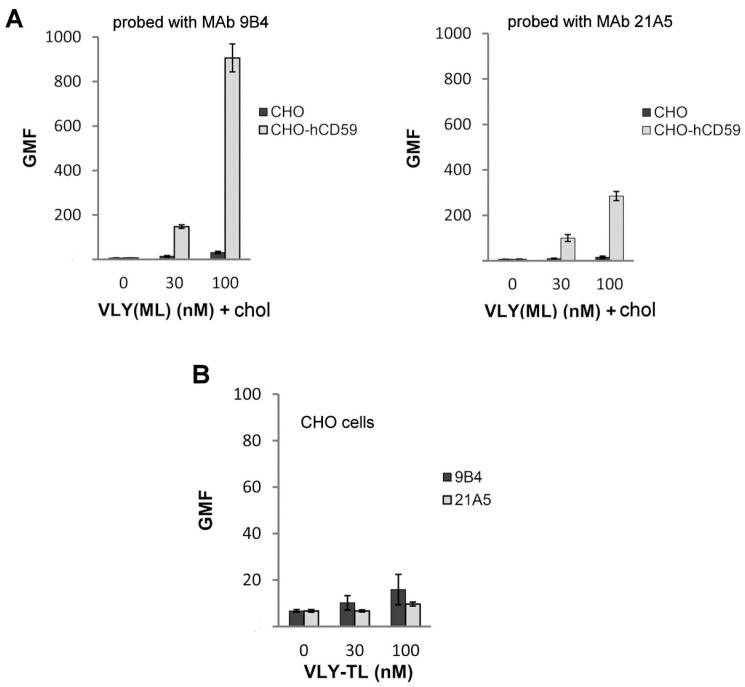
(**A**) Binding of vaginolysin mutant protein VLY(ML) preincubated with cholesterol to CHO and CHO-hCD59 cells probed with monoclonal antibodies (MAbs) 9B4 and 21A5; (**B**) Binding of VLY-TL mutant with impaired cholesterol recognition motif to CHO cells detected with 9B4 and 21A5 MAbs. The figures are representative of experiments performed at least in triplicate. GMF: geometric mean fluorescence.

We preincubated VLY(ML) with cholesterol and assayed for cell binding. Binding of cholesterol-preincubated VLY(ML) to CHO cells was not detected by flow cytometry, however binding to CHO-hCD59 cells was retained (Figure 4A). Mutant protein VLY-TL with the impaired cholesterol recognition site did not bind to CHO cells (Figure 4B).

### 2.3. VLY Oligomer Formation

**Figure 5:**
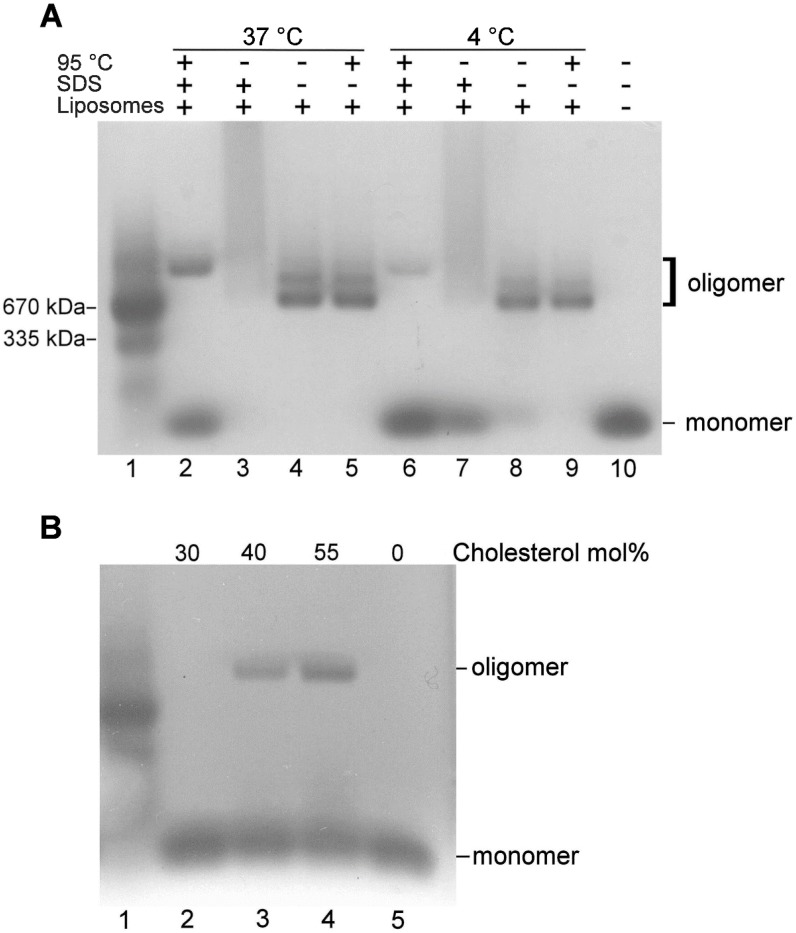
Oligomer formation on cholesterol-rich liposomes by vaginolysin (VLY) detected by denaturing agarose gel electrophoresis. (**A**) Native VLY was incubated with the cholesterol-rich liposomes and the resulting oligomer species were separated on SDS-agarose gel; (**B**) Denaturing agarose gel electrophoresis of VLY oligomers formed on cholesterol-rich liposomes with varying concentrations of cholesterol embedded in liposomes. Lane 1 in panels (**A**), (**B**): molecular mass marker bovine thyroglobulin crosslinked with glutaraldehyde. The protein prepared in SDS-containing sample buffer was heated to 95 °C for 7 min. Thyroglobuline forms a dimer of ~670 kDa and a monomer (~335 kDa) is also observed.

The formation of ILY oligomers was observed on cholesterol-rich human cell membranes [13]. However, it was reported that ILY binds well to cholesterol-rich liposomes lacking hCD59, although this interaction does not induce ILY structural changes leading to oligomerization with subsequent β-barrel pore formation [26]. This suggests that cholesterol does not promote β4-β5 disruption enabling ILY monomers to oligomerize via the interaction of adjacent β1 and β4 strands [26]. We examined whether VLY could form an oligomeric complex on cholesterol-rich membranes that lack hCD59. In parallel, we also assayed ILY (hCD59-dependent) and pneumolysin (PLY) (hCD59-independent) oligomerization on liposomes. VLY was incubated with the cholesterol-rich DOPC liposomes and subjected to SDS-agarose electrophoresis (SDS-AGE), which can separate monomeric and large oligomeric complexes of the CDCs [27]. In the absence of liposomes VLY migrates as a monomer on the SDS-agarose gel (Figure 5A, lane 10). Incubation of VLY with liposomes at 37 °C resulted in the formation of oligomeric VLY complexes (Figure 5A, lanes 2–5). VLY oligomers were also formed at 4 °C but apparently in smaller quantities (Figure 5A, lanes 6–9). The stability of the oligomeric complex was examined by subjecting VLY-liposome mixtures to SDS and heat treatment. The oligomeric non-homogeneous complexes were formed without either SDS in the sample buffer or sample heat treatment or without any treatment of the sample (Figure 5A, lanes 3–5 and 7–9). Heating the SDS-treated VLY-liposome mixture resulted in dissociation of about 50% of the complexes into the monomers, but the remaining VLY formed a homogeneous oligomer of higher molecular mass (Figure 5A, lanes 2, 6). The VLY oligomer was also obtained using cholesterol-rich POPC liposomes (data not shown). We did not observe any VLY dimers either in the presence or in the absence of liposomes. The VLY oligomer band exhibited slower migration on the gel than the high molecular mass marker band of 670 kDa (Figure 5A, lane 2). Like in other studies [27] we were not able to estimate the molecular mass of VLY oligomer on SDS-agarose gel. We did not observe ILY oligomerization on cholesterol-rich liposomes (Figure S2), while heating of SDS-treated PLY-liposome mixture resulted in the formation of a homogeneous oligomer. Moreover, we also observed formation of the PLY dimer in the absence of liposomes (Figure S2).

Non-hemolytic VLY(ML) and VLY-TL mutants did not form oligomers on cholesterol-rich liposomes (Table 1). The explanation of the failure to oligomerize is different in each case. In VLY(ML) the disengagement of β5 from β4 is presumably blocked by the substitution of glycines thus trapping the mutant protein in the monomer-locked state and freezing the conversion of monomers into oligomers [3]. The VLY-TL mutant lacks the intact cholesterol-recognition motif [9] and as a result is not able at all to bind the membrane-immobilized cholesterol or the cholesterol embedded in liposomes.

The assembly of oligomeric complexes of native VLY was dependent on cholesterol concentration in DOPC liposomes. Oligomeric complexes identified by SDS-AGE were formed on the liposomes with the cholesterol concentration in the range of 40–55 mol % (Figure 5B). Overall, our studies of VLY interaction with liposomes demonstrated that cholesterol is able to promote VLY structural changes leading to oligomerization.

### 2.4. Analysis of VLY Monomer-Monomer Interface

**Figure 6:**
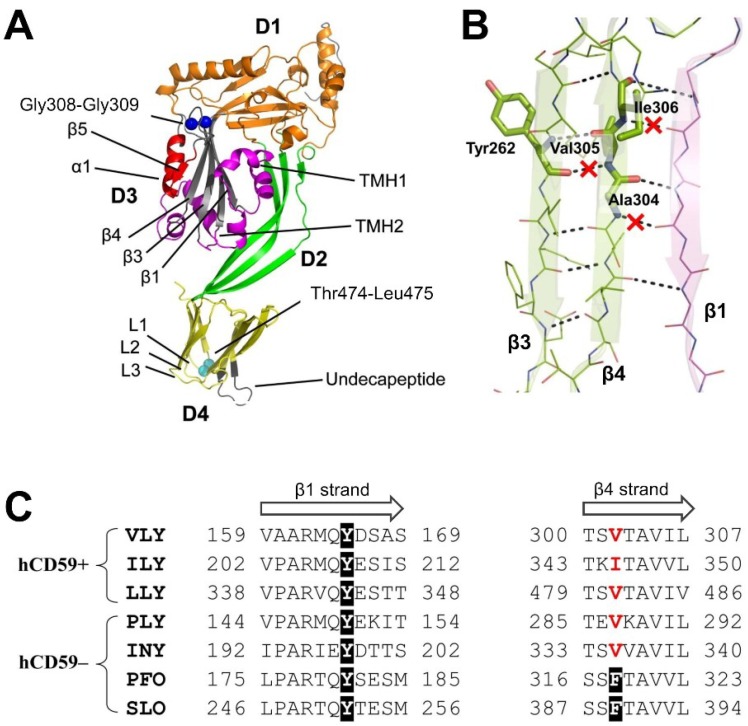
(**A**) Molecular model of vaginolysin (VLY). Domains are designated as D1-D4; TMH1-TMH2 motifs—purple ribbons; the double Gly motif is shown as dark blue space-filled spheres; the Thr-Leu pair in D4 is shown as light blue spheres; (**B**) A model of interaction between β1 and β4-strands of the neighboring VLY monomers. Hydrogen bonds disrupted as a result of proline substitutions at positions 304–306 in β4 are indicated with red crosses; (**C**) Multiple sequence alignment of β1 and β4 strands of several CDCs, whose toxic activity is hCD59 dependent (hCD59+) or independent (hCD59–). Abbreviations for cholesterol-dependent cytolysins: VLY, vaginolysin; ILY, intermedilysin; LLY, lectinolysin; PLY, pneumolysin; INY, inerolysin; PFO, perfringolysin; SLO, streptolysin O. UniProt accession numbers: VLY, B2ZUI3; ILY, Q9LCB8; PLY, Q04IN8; SLO, P0DE96; PFO, P0C2E9. Other sequence sources are as follows: INY, ZP_05744302 (NCBI); LLY [30].

Most of the knowledge regarding regions involved in oligomerization of CDCs comes from studies of perfringolysin O (PFO) [3,28]. It is believed that the central PFO monomer-monomer interface is both stabilized and properly aligned due to the π-stacking interaction between Tyr-181 and Phe-318 in the respective β1 and β4 strands of neighboring monomers [3]. However, several CDCs including VLY, pneumolysin and ILY, lack one of the corresponding aromatic residues in the motifs that constitute the presumed monomer-monomer interface (Figure 6C). We, therefore, asked whether Tyr-165, one of the preserved aromatic residues in β1 of VLY, may still be important for interface formation. To our surprise, substitution of Tyr-165 with Ala essentially had no effect (Table 1), raising a question whether VLY oligomerizes through the same polypeptide regions (β1 and β4 strands) as PFO. We decided to test that by preventing the possibility of edge-to-edge interaction between β-sheets of the neighboring monomers. Using the homology model of VLY (Figure 6A), based on the closely related (58% sequence identity) crystal structure of ILY [29], we identified β4 residues that could potentially contribute their NH-groups to the hydrogen bond formation with β1 of the neighboring monomer (Figure 6B). We then targeted two of those residues, Ala-304 and Ile-306, for substitution with proline.

The rationale for proline substitutions was based on the fact that proline lacks the NH-group needed to maintain the hydrogen-bonding pattern with the neighboring β-strand. The NH-groups of Ala-304 and Ile-306 in the VLY model are oriented towards the β5 strand and after its rotation would be expected to become accessible for pairing with β1 of another monomer. We constructed two VLY mutants, I306P and A304P·I306P, that remove one and two NH-groups, respectively (Figure 6B). Both gave a non-hemolytic phenotype and lost the ability to form oligomers on cholesterol-rich liposomes (Figure S3, Table 1). At the same time, they both retained the ability to bind to membrane-immobilized cholesterol (Figure S3A) suggesting that these mutations did not alter the overall structure of a VLY monomer. To make sure that the mutations in the β4 strand affected only the interface formation, we also constructed and tested the V305P mutant. In contrast to Ala-304 and Ile-306, the NH-group of Val-305 forms a hydrogen bond with tyrosine (Tyr-262) residing in β3 of the same molecule (Figure 6B). When tested, this mutation did not change hemolytic and oligomer-forming properties of VLY (Figure S3, Table 1). Taken together our results indicate that the introduced proline substitutions do not alter the structure of VLY monomer, and that hydrogen bonds formed by Ala-304 and Ile-306 situated in β4 are part of the monomer-monomer interface. In other words, similarly to PFO, VLY oligomers are formed by edge-to-edge pairing of β-sheets from neighboring monomers. However, no π-stacking interaction is needed for either achieving the stability of VLY oligomers or the alignment of β1 and β4 in the proper register relative to each other.

## 3. Discussion

High VLY specificity to human cells and its amino acid sequence relatedness to ILY supported the idea that the cytolytic activity of VLY strictly depends on the complement regulatory protein human CD59 [18,28]. However, our recent study on the reconstitution of VLY into artificial tethered bilayer membranes (tBLMs) lacking hCD59 on their surface demonstrated that the formation of water-filled pores in the membranes was consistent with the exposure of tBLMs to VLY solutions [24]. Preincubation of VLY with neutralizing anti-VLY MAbs and application of cytolytic/hemolytic inactive VLY mutants completely abolished membrane damage. These results suggested that cholesterol plays a decisive role in VLY-mediated pore formation [24].

The study herein demonstrates that VLY binds to CHO cells that do not express hCD59 on their surface. The impairment of VLY cholesterol recognition site either by preincubation with cholesterol or mutation of Thr-Leu pair in Loop L1 of D4 domain (VLY-TL mutant) abolished VLY binding to CHO cells. However, we show that VLY mutant arrested in monomer state (VLY(ML)) with impaired cholesterol recognition site retains binding to hCD59-containing CHO cells. VLY forms SDS and heat-stable oligomers on cholesterol-rich liposomes that lack hCD59. Knockout of the VLY cholesterol recognition site or restriction of the movement of the β5 strand in domain D3 by the respective mutations prevented VLY oligomerization on cholesterol-rich liposomes. We demonstrate that VLY oligomerization on liposomes is dependent on cholesterol concentration. The sharp transition from undetectable binding at less than 40 mol % total membrane cholesterol to maximal binding at 55 mol % is characteristic for CDCs [8,28]. Thus, our data demonstrate that cholesterol alone is sufficient to initiate structural rearrangements in VLY that lead to oligomerization of membrane-bound monomers.

Results of site-directed mutagenesis within the β4 strand of the inferred VLY structure clearly showed that the formation of VLY oligomers on liposomes follows commonly accepted typical steps in CDCs pore-forming mechanism—disruption of β4–β5 interaction in D3 followed by β4-strand association with β1 of another VLY molecule [3,28]. On the other hand, it is still not obvious how the β1–β4 alignment is locked in the proper register and how the interaction stability between VLY monomers is achieved. What is obvious, however, is that the π-stacking interaction proposed for PFO [3,28] has no role in VLY. Firstly, only one of the two corresponding aromatic residues (Tyr-165) is present, and secondly, its substitution has no effect on the formation of an oligomeric VLY complex. Inspection of other CDC sequences (Figure 6C) and their homologs revealed that many of them, similarly to VLY, do not maintain the corresponding pair of aromatic residues. Therefore, high-resolution experimental structure of oligomeric form of VLY or of its homologs would be most helpful for detailed understanding of interactions that define the monomer-monomer interface.

It is likely that VLY may act following two alternative pathways: one is similar to most CDCs (e.g., PFO) where binding to cholesterol initiates changes in the monomer structure that lead to the formation of the pore complex [3,28]. The second pathway is characteristic for ILY where cholesterol is not involved in membrane recognition, but binding to hCD59 initiates assembly of the prepore complex and cholesterol is important for the prepore conversion to pore [9,12,16,28]. Recently, it was reported that Sm-hPAF (a homolog of lectinolysin) from *Streptococcus mitis*, similarly to VLY, exhibits affinity to both cholesterol and hCD59 [23]. Our data on VLY cytolytic activity thus support the attribution of VLY together with Sm-hPAF to a novel group (group III) of atypical CDCs in the classification based on the mode of receptor recognition introduced Tabata *et al.* [23]. It has been suggested that CDCs preferentially bind to cholesterol and sphingolipid-enriched membrane microdomains known as lipid rafts [31,32,33]. Other studies demonstrated that exposure of cholesterol and toxin binding are not limited to the presence of the particular membrane domain [8,34,35]. Despite previous efforts, the molecular basis for CDCs-cholesterol binding still remains enigmatic. Results of the current study allow us to hypothesize that the increased effectiveness of lysis of human cells might be connected with the GPI-anchored hCD59 association with lipid rafts [36,37]. Human CD59 located within lipid rafts [36,37] is a prime candidate to attract VLY molecules and increase the possibility of hCD59-attached VLY molecules to encounter each other thereby accelerating and facilitating VLY oligomerization. It could be that hCD59 recruits VLY molecules increasing local monomer VLY concentration needed for toxin oligomerization on the membranes. VLY concentration in the human body is low; therefore VLY is forced to act in the most effective, hCD59-dependent way to induce cell damage.

## 4. Experimental Section

### 4.1. Generation of Vaginolysin Mutants

Recombinant *N*-terminally-hexahistidine-tagged VLY lacking the putative signal sequence (amino acid (aa) residues 1–31) was expressed and purified as described previously [21]. All VLY mutants were generated using pUC57 plasmid (Thermo Fisher Scientific, Vilnius, Lithuania) carrying the VLY-coding gene lacking aa 1–31 as a template for PCR-mediated, site-directed mutagenesis targeted to the whole plasmid. All PCR generated VLY-encoded sequences were confirmed by DNA sequence analysis. The genes coding VLY mutant proteins were cloned into pET28a(+) vector (Novagen/Merck KGaA, Darmstadt, Germany) for construction of hexahistidine-tagged proteins. VLY mutants were expressed in *E. coli* and purified as described previously [21]. Purified recombinant proteins were stored in 20 mM sodium acetate (pH5.5) (Merck KGaA, Darmstadt, Germany), 0.1 M (NH_4_)_2_SO_4_ (Merck KGaA, Darmstadt, Germany), 0.01% Tween-80 (Carl Roth, Karlsruhe, Germany) buffer.

### 4.2. Generation of Pneumolysin and Intermedilysin

The DNA fragment of 1503 bp containing ILY coding sequence was amplified from *Streptococcus intermedius* isolate 14654 (kind gift of Lithuanian National Public Health Surveillance Laboratory (LNPHSL), Vilnius, Lithuania) using Pfu proofreading polymerase (Thermo Fisher Scientific, Vilnius, Lithuania). The PCR fragment was sequenced and deduced amino acid sequence revealed 99% identity to ILY from *S. intermedius* ATCC 27335. PCR product comprising ILY coding gene lacking the putative signal sequence (aa residues 1–33) was cloned into pET28a(+) vector (Novagen/Merck KGaA, Darmstadt, Germany) and transformed into *E. coli* BL21(DE3) strain (Merck KGaA, Darmstadt, Germany). Recombinant *N*-terminally-hexahistidine-tagged ILY was purified from 2 L of culture using affinity chromatography on 6BCL-IDA Ni-sepharose (GE Healthcare, Helsinki, Finland) and ion-exchange chromatography on SP sepharose FF (GE Healthcare, Helsinki, Finland) according to manufacturer’s recommendations. Purified recombinant protein was stored in 20 mM sodium acetate (pH5.5) (Merck KGaA, Darmstadt, Germany), 0.1 M (NH_4_)_2_SO_4_ (Merck KGaA, Darmstadt, Germany, 0.01% Tween-80 (Carl Roth, Karlsruhe, Germany) buffer.

The DNA fragment of 1416 bp containing pneumolysin (PLY) coding sequence was amplified from *Streptococcus pneumoniae* isolate 7170 (kind gift of LNPHSL). The gene for PLY was sequenced and cloned into pET28a(+) vector. Expression and purification of PLY were carried out as described for ILY. Purified PLY was stored in 20 mM HEPES (pH 7.5) (Merck KGaA, Darmstadt, Germany), 0.2 M NaCl (Merck KGaA, Darmstadt, Germany) buffer.

### 4.3. Determination of VLY and ILY Hemolytic Activity

Hemolytic activity of each VLY mutant as well as native VLY and ILY was determined on human erythrocytes as described previously [21]. PBS supplemented with 0.001% Tween-80 (Carl Roth, Karlsruhe, Germany) was included as a negative control. The HD_50_ was defined as the concentration of cytolysin required to lyse 50% of human or mouse erythrocytes and was determined by hemolytic assay performed in triplicate.

### 4.4. Determination of VLY Cytolytic Activity

Adherent human epitheloid HeLa cells (ATCC (Rockville, MD, USA) Cat. No. CCL-2) were cultivated in Dulbecco’s Modified Eagle Medium (DMEM) (Biochrom, Berlin, Germany) supplemented with 10% fetal bovine serum (FBS) (Biochrom, Berlin, Germany) and 100 µg/mL gentamicin (Carl Roth, Karlsruhe, Germany). Chinese hamster ovary cells (CHO) (ECACC (Salisbury, UK) Cat. No. 85050302) were cultivated in 1:1 mixture of DMEM (Biochrom, Berlin, Germany) and Nutrient Mixture F-12 (Gibco by Life Technologies, Grand Island, NY, USA) (DMEM:F-12) medium supplemented with 10% FBS. The cells were grown at 37 °C and 5% CO_2_ in 96-well plates to approximately 70% confluence. After removing growth medium, the cell monolayer was rinsed twice with serum-free DMEM. Recombinant VLY was diluted in serum-free DMEM to yield concentrations of VLY ranging from 1.1 to 250 nM. One-hundred µL of each VLY mixture were added to the wells with HeLa, CHO and CHO-hCD59 cells and the plates were incubated for 1 h at 37 °C and 5% CO_2_. As a negative control, reaction mixture without VLY and cell-free DMEM supplemented with 0.001% Tween-80 (Carl Roth, Karlsruhe, Germany) without VLY were used. After incubation, cell viability was determined by a colorimetric assay using 3-(4,5-dimethylthiazol-2-yl)-5-(3-carboxymethoxyphenyl)-2-(4-sulfophenyl)-2H-tetrazolium (MTS) staining. Twenty µL of ready-to-use MTS solution (Promega, Madison, WI, USA) was added to the wells and the plates were incubated for 2 h at 37 °C and 5% CO_2_. The optical density (OD) was measured at 490/630 nm wavelengths in a microplate reader (Thermo Scientific Multiskan Go, Vantaa, Finland). Cytotoxicity assay measurements were performed in triplicate. Cell viability was also assessed microscopically at magnifications 20× and 40× using microscope Olympus IX70 (Olympus, Tokyo, Japan).

### 4.5. Generation of CHO Cell Line Expressing hCD59

The Fc coding sequence in mammalian expression vector pFUSE-hIgG1-Fc2 (InvivoGen, San Diego, CA, USA) was cut off using EcoRI and NheI restriction endonucleases (both enzymes were purchased from Thermo Fisher Scientific, Vilnius, Lithuania) and synthetic hCD59 coding gene (GenScript, Piscataway, NJ, USA) comprising amino acids 1–76 and GPI-anchoring sequence at the 3'-terminal end of the gene were inserted. The glycosylation site Asn-18 of hCD59 was changed to Gln. The removal of a single glycosylation site does not change the hCD59 activity [38]. The resulted plasmid pFUSE-hCD59 bearing Zeocin-resistance gene *Sh ble* was transfected into Chinese hamster ovary cells (CHO) using TurboFect reagent (Thermo Fisher Scientific, Vilnius, Lithuania). The cells were cultivated in DMEM:F-12 (1:1) medium supplemented with 10% FBS (Biochrom, Berlin, Germany) and 250 µg/mL Zeocin (InvivoGen, San Diego, CA, USA). Positive clones were selected by flow cytometry using FITC-conjugated Mouse Anti-Human CD59 antibodies (BD Pharmingen, Franklin Lakes, NJ, USA). An empty (control) vector pFUSE-hIgG1 was obtained by fill-in of EcoRI and NheI 5'-overhangs with subsequent blunt ends ligation. CHO cells were transfected with the control vector and grown as described above.

### 4.6. Flow Cytometry

Subconfluent CHO and CHO-hCD59 cells were detached with StemPro Accutase (Gibco by Life Technologies, Grand Island, NY, USA), washed and resuspended in PBS. Forty microliters of cell suspension (1 × 10^5^ cells) were mixed with 10 µL of each VLY mutant (diluted in PBS) to the final concentrations of 30 and 100 nM. Native VLY was used as a positive control. Reaction mixture without VLY was used as a negative control. Where noted, VLY mutants were preincubated with cholesterol (Sigma-Aldrich, St. Louis, MO, USA) (1 μL of 1.6 mg/mL cholesterol in chloroform was added to 9 μL of toxin) or control (chloroform alone at the corresponding dilution) at 37 °C for 10 min prior to cell treatment. The reaction mixture was incubated at 37 °C for 30 min and washed with incubation buffer (PBS supplemented with 1% FBS and0.1% NaN_3_ (Sigma-Aldrich, St. Louis, MO, USA)). Then the cells were incubated with an excess of primary anti-VLY MAbs (either 9B4 or 21A5 [21]) prepared in the same incubation buffer for 30 min at room temperature (RT). After washing, cells were incubated with an excess of secondary PE-conjugated Goat Anti-Mouse IgG antibodies (BD Pharmingen, Franklin Lakes, NJ, USA) in incubation buffer at RT for 30 min. After washing, the cells were fixed using BD CytoFix (BD Biosciences, San Jose, CA, USA) at 4 °C for 10 min and after washing transferred to 200 µL of incubation buffer and read by CyFlow space flow cytometer (Partec, Muenster, Germany). Flow cytometry data were analyzed using FCS Express 4 software (Version 4.07.0020, De Novo Software, Los Angeles, CA, USA, 2014), gating on live cells (after cell fixation the distribution of live/dead cells was determined). All incubations with the native VLY were performed at 4 °C in order to avoid cell lysis, however the cytolysis was not evaded. The main fraction of cells was lysed, therefore the data obtained with the native VLY and cell binding were analyzed without gating. The geometric mean fluorescence intensity was used to evaluate the binding of VLY and its derivatives to CHO and CHO-hCD59 cells.

### 4.7. Indirect ELISA

The apparent dissociation constants (*K*_d_) of MAbs 9B4 and 21A5 were determined by an indirect ELISA. Microtiter plates (Nunc MaxiSorp, Nunc, Roskilde, Denmark) were coated with recombinant purified VLY and its derivatives by adding 100 μL of VLY solution (5 μg/mL) in coating buffer (50 mM Na-carbonate, pH 9.5 (Carl Roth, Karlsruhe, Germany)) and incubated overnight at 4 °C. Plates were blocked for 1 h at RT with 1% BSA (PAA Laboratories GmbH, Pasching, Austria) in PBS and then incubated with 100 μL serially diluted anti-VLY MAbs 9B4 and 21A5 (concentration ranged from 1.9 × 10^−7^ to 3.3 × 10^−12^ M in PBS with 0.1% Tween-20 (Carl Roth, Karlsruhe, Germany)) for 1 h at RT. After washing, plates were incubated with HRP-conjugated Goat Anti-Mouse IgG (Bio-Rad, Hercules, CA, USA) (diluted 1:5000 in PBS with 0.1% Tween-20) at RT for 1 h. After washing, the enzymatic reaction was developed with TMB substrate (Sigma-Aldrich, St. Louis, MO, USA) and stopped by adding 1 M H_2_SO_4_ (Sigma-Aldrich, St. Louis, MO, USA). The optical density was measured at 450 nm (OD_450_) in a microtiter plate reader (Thermo Scientific Multiskan Go, Vantaa, Finland). The *K*_d_ for each MAb was defined as the concentration of MAb that gives one-half of the maximum OD_450_ value.

### 4.8. Preparation of Liposomes

Vesicles were prepared using 0.001 mol/L chloroform solutions of cholesterol/DOPC and cholesterol/POPC at a molar ratio of 30/70, 40/60 and 55/45. 1,2-dioleoyl-*sn*-glycero-3-phosphocholine (DOPC) and 1-palmitoyl-2-oleoyl-*sn*-glycero-3-phosphocholine (POPC) were purchased from Avanti Polar Lipids, Alabaster, AL, USA. A lipid film was prepared by evaporating 1 mL of the chloroform solution in a gentle stream of nitrogen followed by a vacuumdrying for 1–2 h. The lipid film was re-dissolved in 1 mL of pentane and dried overnight. The film was hydrated by adding 1 mL of working buffer, 0.1 mol/L NaCl (Merck KGaA, Darmstadt, Germany), 0.5 × 10^−3^ mol/L NaH_2_PO_4_ (Carl Roth, Karlsruhe, Germany) (pH 7.4), sonicated for 60 min, and incubated with occasional vortexing, as needed until the lipid film was no longer visible. The lipid preparation was then extruded 21 times through a 100 nm polycarbonate membrane (Avanti Polar Lipids, Alabaster, AL, USA). Vesicle size (~50–100 nm) was determined by dynamic light scattering as described earlier [39]. The prepared liposomes were stored at 4 °C and used within seven days of preparation.

### 4.9. Cholesterol Dot Blot Analysis

Cholesterol (Sigma-Aldrich, St. Louis, MO, USA) was dissolved and serially diluted in chloroform to concentrations ranging from 32.5 to 0.01 mM (*w*/*v*). Polyvinylidene Roti-PVDF membrane (pore size 0.45 µm) (Carl Roth, Karlsruhe, Germany) was wetted with 100% methanol (Carl Roth, Karlsruhe, Germany) and washed in PBS buffer. Dots with different amounts of immobilized cholesterol were formed by loading 2 µL of each cholesterol (Sigma-Aldrich, St. Louis, MO, USA) solution on the membrane. The membrane was dried for 30 min and blocked with 3% low fat powered milk (Carl Roth, Karlsruhe, Germany) (dissolved in PBS) for 1 h at RT. Then the membrane was incubated with VLY and its derivatives in blocking solution with 0.01% Tween-20 (Carl Roth, Karlsruhe, Germany) for 1 h at RT. Blots were washed with washing solution (PBS with 0.01% Tween-20) and incubated with anti-VLY MAb 9B4 in blocking solution with 0.01% Tween-20 for 1 h at RT. After washing, the blots were incubated with HRP-conjugated goat anti-mouse IgG antibodies (Bio-Rad, Hercules, CA, USA) (1:5000 in blocking solution with 0.01% Tween-20) for 1 h at RT. After washing, blots were incubated with 3,3',5,5'-tetramethylbenzidine (TMB) Liquid Substrate System for Membranes (Sigma-Aldrich, St. Louis, MO, USA) for 1 min and the reaction was stopped by immersing the membrane into water.

### 4.10. Denaturing Agarose Gel Electrophoresis

Denaturing agarose gel electrophoresis (SDS-AGE) was carried out as described earlier [27]. Briefly, agarose (TopVision LE GQ Agarose, Thermo Fisher Scientific, Vilnius, Lithuania) was diluted in Tris-Glycine SDS buffer (Thermo Fisher Scientific, Rockford, IL, USA) to obtain final concentration of 1.5% (*w*/*v*). VLY and its derivatives (53.7 pmol) were incubated alone or with liposomes (15 nmol of total lipid) for 30 min at 37 °C or 4 °C. PLY (54.6 pmol) and ILY (52.4 pmol) were incubated either alone or with liposomes for 30 min at 37 °C and subjected to SDS-AGE as described for VLY. The sample buffer (with or without 2% (*w*/*v*) SDS) (Sigma-Aldrich, St. Louis, MO, USA) was added to reaction mixture and the mixture was incubated for 2 min at 37 °C. Before loading the samples were heated for 7 min at 95 °C unless otherwise stated. The gel was stained in the solution comprised of 0.25% (*w*/*v*) Coomassie Brilliant Blue R (Sigma-Aldrich, St. Louis, MO, USA), 10% (*v*/*v*) acetic acid and 40% (*v*/*v*) methanol (both reagents from Carl Roth, Karlsruhe, Germany). The destaining solution contained 5% (*v*/*v*) acetic acid and 20% (*v*/*v*) methanol.

### 4.11. Construction and Assessment of the Homology-Based VLY Structural Model

VLY homology model was constructed with HHpred [40] using the crystal structure of ILY [29] (PDB id: 1S3R) as the closest structural template (58% sequence identity). To have an additional reference point, we also constructed a homology model of perfringolysin O (PFO) using the same ILY structure as the modeling template (in this case sharing 41% identical residues). Since the experimental structure of PFO is known [41], the model was constructed using the “ideal” (structure-based) alignment between PFO and ILY. Assessment of structure quality was performed using Prosa knowledge-based energy potentials [42]. More negative Prosa Z-scores indicate more energetically favorable structures. Prosa Z-score of the VLY model (−11.31) is intermediate between those of the ILY structure (−12.27) and PFO model (−11.08) suggesting that VLY structurally is closer to ILY than PFO, and that the accuracy of VLY model is sufficient to provide details at the level of individual residues.

### 4.12. Ethics Statement

The use of human erythrocytes from healthy adult volunteer followed written informed consent was approved by the Council of the Institute of Biotechnology, Vilnius University (Protocol of 20/11/2013, no. 54).

## Supplementary Materials

Supplementary materials can be accessed at: http://www.mdpi.com/2072-6651/7/1/0110/s1.
